# Carotid Duplex-Derived Markers Across Angiographic Coronary Artery Disease Burden: A Pandemic-Era Real-World Cohort Study †

**DOI:** 10.3390/jcm15114383

**Published:** 2026-06-05

**Authors:** Tuna Aras, Armine Grigorian, Mahmoud Tayeh, Adel Aswad, Mohamed Sharkawy, Zaki Almuzakki, Bernhard Dorweiler, Grigore Cernaianu, Payman Majd

**Affiliations:** 1 EVK Bergisch Gladbach, Department of Vascular Surgery, Ferrenbergstraße 24, 51465 Bergisch Gladbach, Germany; 2 BG Klinikum Duisburg gGmbH, Großenbaumer Allee 250, 47249 Duisburg, Germany; 3 Al-Qassimi Teaching Hospital and Cardiac Centre, University of Sharjah, Sharjah P.O. Box 3500, United Arab Emirates; 4 Cairo University Hospitals, Cairo 4240310, Egypt; 5 Department of Vascular and Endovascular Surgery, Faculty of Medicine and University Hospital of Cologne, University of Cologne, 50937 Cologne, Germany; 6 Division of Pediatric Surgery, Faculty of Medicine and University Hospital of Cologne, University of Cologne, Kerpenerstr. 62, 50937 Cologne, Germany

**Keywords:** carotid stenosis, duplex ultrasonography, peak systolic velocity, coronary artery disease, coronary angiography, systemic atherosclerosis, polyvascular disease, COVID-19 pandemic, carotid-index

## Abstract

**Background:** Carotid atherosclerosis is a recognised manifestation of systemic vascular disease, and its association with coronary artery disease (CAD) has been well described. However, previous studies have largely been conducted under conventional diagnostic conditions and have focused on carotid plaque, intima–media thickness, or simple present-versus-absent stenosis classifications, rather than duplex-derived haemodynamic markers across the spectrum of angiographic CAD burden. The COVID-19 pandemic and post-pandemic period changed referral patterns and created more variable cardiovascular presentations, including symptoms that could resemble or mask obstructive CAD. Therefore, we investigated whether the established association between carotid stenosis severity and CAD burden remains detectable in a diagnostically heterogeneous real-world cohort, and whether routinely available carotid duplex haemodynamic parameters provide a clinically relevant signal in this setting. **Methods:** This single-centre, cross-sectional study was performed as a carotid-focused secondary analysis of the BG Study cohort. We included 902 consecutive patients who underwent invasive coronary angiography between 2021 and 2023 and carotid duplex ultrasonography during the same hospitalisation. CAD burden was defined according to the number of major coronary vessels with ≥70% diameter stenosis and classified as no CAD, one-vessel, two-vessel, or three-vessel disease. Carotid duplex parameters included peak systolic velocities of the common, internal, and external carotid arteries, as well as ICA stenosis severity graded according to NASCET criteria. Associations with CAD burden were assessed using a staged statistical approach combining χ^2^ tests, Kruskal–Wallis tests with post hoc pairwise comparisons, Spearman correlation, inverse probability weighting, and ordered logistic regression. **Results:** The prevalence of measured ICA stenosis of any grade and severe ICA stenosis increased with greater CAD burden (both *p* < 0.001). Median PSV values of the bilateral ICAs and ECAs differed significantly across CAD groups on global intergroup testing. Post hoc pairwise analyses showed that significant corrected differences were concentrated between patients without CAD and those with multivessel or three-vessel CAD, particularly for ICA stenosis measures and bilateral ECA PSV. Spearman analysis demonstrated weak but statistically significant correlations between carotid parameters and CAD burden (ρ = 0.085–0.134). After inverse probability weighting, covariate balance was achieved, with all post-IPW standardised mean differences being <0.01. In ordered logistic regression (OLR) analysis, patient-reported history of carotid stenosis (OR 2.25, 95% CI 1.38–3.67; *p* < 0.001), right external carotid artery PSV per 10 cm/s (OR 1.31, 95% CI 1.09–1.57; *p* = 0.004), left ICA PSV per 10 cm/s (OR 1.17, 95% CI 1.01–1.36; *p* = 0.034), and left ICA stenosis per 10% (OR 1.24, 95% CI 1.11–1.39; *p* < 0.001) were independently associated with higher CAD burden. Exploratory ratio-based analyses showed that the ECA/CCA PSV ratio was associated with CAD presence and higher CAD burden, whereas the ICA/CCA ratio showed weaker associations; neither ratio-based index outperformed absolute ECA PSV. **Conclusions:** In this carotid-focused secondary analysis of a pandemic-era angiography cohort, carotid stenosis severity and duplex-derived haemodynamic parameters were independently but modestly associated with increasing angiographic CAD burden. These findings support carotid duplex markers as adjunctive indicators of systemic atherosclerotic burden rather than standalone tools for CAD detection or treatment decision-making. Future validation in vascular surgery populations is warranted to determine whether routinely available carotid duplex parameters can contribute to targeted cardiovascular risk recognition before major vascular procedures.

## 1. Introduction

Patients undergoing vascular evaluation frequently carry a high burden of systemic atherosclerosis and may harbour clinically silent coronary artery disease (CAD). Although routine extensive cardiac testing is not recommended for all patients with stable symptoms, selected vascular patients present with advanced cardiovascular risk profiles and may have unrecognised CAD or silent coronary ischaemia. In such patients, occult coronary disease may contribute substantially to peri-operative and long-term morbidity and mortality, particularly through major adverse cardiac events (MACEs).

Carotid duplex ultrasonography is widely used to assess extracranial cerebrovascular disease and guide the management of carotid stenosis. In Germany and similar vascular surgery practice settings, carotid duplex is often performed directly within the vascular specialist assessment. Therefore, duplex findings may provide immediately available information beyond revascularisation eligibility, including potential signals of broader systemic atherosclerotic burden. This is clinically relevant because vascular surgery patients frequently have multiterritorial atherosclerosis; may undergo major procedures associated with blood loss, haemodynamic stress, and systemic inflammatory activation; and may have cardiovascular risk that is under-recognised or not optimally treated before major vascular surgery. Symptoms such as chest pressure, dyspnoea, claudication, or reduced walking capacity may be underreported, normalised, or attributed to ageing, musculoskeletal disease, or the known peripheral vascular condition. Consequently, clinically relevant CAD or silent coronary ischaemia may remain undetected until the peri-operative period or later follow-up.

The association between carotid atherosclerosis and CAD has been described previously. However, much of the existing literature has focused on carotid plaque, intima–media thickness, or simple present-versus-absent stenosis classifications. Less is known about whether duplex-derived haemodynamic parameters, particularly internal and external carotid artery peak systolic velocities, are associated with angiographic CAD burden across no-, one-, two-, and three-vessel disease.

A distinctive feature of the BG Study [1] cohort is its recruitment during the COVID-19 pandemic and post-pandemic period, when altered referral pathways and heterogeneous cardiovascular symptom profiles may have increased diagnostic uncertainty. This provided a stress-test setting for the carotid–coronary association: if carotid duplex markers remained associated with CAD burden in this cohort, the observed signal would likely reflect a robust systemic atherosclerotic relationship.

## 2. Methods

### 2.1. Study Design and Population

This single-centre, cross-sectional study was performed as a carotid-focused secondary analysis of prospectively collected data from the BG Study cohort. The cohort included patients admitted to the Department of Cardiology, EVK Bergisch Gladbach, Germany, between 2021 and 2023 for evaluation of coronary artery disease (CAD). All patients underwent invasive coronary angiography and carotid duplex ultrasonography during the same hospitalisation. Carotid duplex ultrasonography was performed by vascular surgery specialists using a Philips Affiniti 30 ultrasound system equipped with an L12-5 linear-array transducer (Philips GmbH Market DACH, Hamburg, Germany). Patients with incomplete coronary angiography or carotid ultrasound data were excluded, resulting in a final cohort of 902 patients.

The primary endpoint was the association between angiographically defined CAD burden and carotid duplex parameters. The study was approved by the Ethics Committee of the Ärztekammer Nordrhein-Westfalen (No. 2020300), and all patients provided written informed consent. The study was conducted in accordance with STROBE guidelines.

### 2.2. Data Collection

CAD burden was defined according to the number of major coronary vessels with ≥70% diameter stenosis on invasive coronary angiography and categorised as no CAD, one-vessel disease, two-vessel disease, or three-vessel disease.

Carotid duplex ultrasonography was performed by vascular surgery specialists using a Philips Affiniti 30 system with a linear array transducer. Standardised examination protocols were applied, including a 60° insonation angle and central sampling of the vessel lumen.

Peak systolic velocity (PSV) was measured in the common carotid artery (CCA), internal carotid artery (ICA), and external carotid artery (ECA). ICA stenosis was classified according to NASCET criteria [2] as mild (<50%), moderate (50–69%), or severe (≥70%). ICA stenosis was analysed both categorically and as a continuous percentage because these approaches provide complementary statistical information. Categorical analysis reflects clinically defined NASCET-based stenosis groups, whereas continuous analysis preserves the full severity gradient and reduces information loss from threshold-based grouping. End-diastolic velocity was not included in the analysis because of collinearity with PSV.

As exploratory sensitivity analyses, ipsilateral ICA/CCA and ECA/CCA PSV ratios were also calculated. Patient-level ratio indices were derived using the maximum bilateral ratio. Total ICA occlusion was excluded because the ICA/CCA ratio is not applicable in complete occlusion.

### 2.3. Statistical Analysis

Statistical analyses were performed using IBM SPSS Statistics v31.0.1.0 (IBM Corp., Armonk, NY, USA) and Python v3.11 (Python Software Foundation, Wilmington, DE, USA). Statistical significance was defined as *p* < 0.05 using two-sided tests. Continuous variables were assessed for normality using histograms, Q–Q plots, and the Shapiro–Wilk test. Because most continuous variables were non-normally distributed, non-parametric methods were used.

A staged statistical approach was used to distinguish descriptive group differences, monotonic CAD-burden gradients, and independently adjusted associations after covariate balancing. Differences across CAD burden groups were assessed using the Kruskal–Wallis test for continuous variables and χ^2^ tests for categorical variables. When global intergroup tests were significant, post hoc pairwise comparisons were performed using Dunn’s test with Holm correction for continuous PSV variables and pairwise Fisher’s exact tests with Bonferroni correction for categorical variables. Associations between carotid parameters and CAD burden were evaluated using Spearman correlation coefficients.

Inverse probability weighting (IPW) was applied using propensity scores derived from logistic regression including age, sex, diabetes mellitus, hypertension, smoking status, LDL cholesterol, creatinine, and eGFR. Covariate balance before and after weighting was assessed using standardised mean differences. Independent associations between carotid parameters and increasing CAD burden were evaluated using ordered logistic regression (OLR), with CAD burden modelled as an ordinal outcome: no CAD, one-vessel disease, two-vessel disease, and three-vessel disease. Multicollinearity was assessed using variance inflation factors, model fit was evaluated using likelihood ratio tests, and the proportional odds assumption was assessed using the Brant test.

Exploratory ratio-based analyses evaluated ICA/CCA and ECA/CCA PSV ratios as supplementary haemodynamic markers. These analyses were performed to determine whether conventional ratio-based carotid indices provided additional information beyond absolute ICA and ECA PSV values.

## 3. Results

A total of 902 patients were included in the analysis. Of these, 310 patients (34.4%) had no coronary artery disease (CAD), 190 (21.1%) had one-vessel disease, 189 (20.9%) had two-vessel disease, and 213 (23.6%) had three-vessel disease. CAD burden was defined using the number of major coronary vessels with ≥70% diameter stenosis. Baseline demographic and clinical characteristics stratified by CAD severity are presented in Table 1.

The prevalence of measured ICA stenosis of any grade differed across CAD categories (*p* < 0.001), increasing from 12.3% in patients without CAD to 28.6% in patients with three-vessel disease. Severe ICA stenosis also increased across CAD categories, from 2.3% in patients without CAD to 12.7% in patients with three-vessel disease (*p* < 0.001). Patient-reported history of carotid stenosis differed across CAD categories (*p* = 0.006), whereas mild and moderate ICA stenosis did not differ significantly. Kruskal–Wallis analysis demonstrated differences in carotid haemodynamic parameters across CAD categories. Left external carotid artery (ECA) peak systolic velocity (PSV) differed between groups (*p* = 0.002), as did right ECA PSV (*p* = 0.017), left ICA PSV (*p* = 0.048), and right ICA PSV (*p* = 0.018). CCA PSV did not differ significantly. These carotid duplex findings are summarised in Table 2.

Post hoc pairwise intergroup comparisons are presented in Table 3. Measured ICA stenosis of any grade differed significantly between patients without CAD and those with one-vessel, two-vessel, and three-vessel CAD after correction for multiple testing. Patient-reported history of carotid stenosis and severe ICA stenosis differed significantly between patients without CAD and those with multivessel CAD, particularly three-vessel disease. Among PSV variables, significant corrected pairwise differences were observed for left and right ECA PSV between patients without CAD and those with three-vessel CAD. No significant corrected pairwise differences were observed for mild or moderate ICA stenosis, and no corrected pairwise PSV differences remained significant for ICA or CCA PSV.

Spearman correlation analysis demonstrated weak but statistically significant associations between carotid parameters and CAD burden. The strongest correlation was observed for left ECA PSV (ρ = 0.134; 95% CI, 0.049–0.217; *p* = 0.002), followed by left ICA stenosis percentage (ρ = 0.113; 95% CI, 0.028–0.196; *p* = 0.002) and right ICA stenosis percentage (ρ = 0.085; 95% CI, 0.023–0.147; *p* = 0.007). All correlations were weak in magnitude.

Inverse probability weighting achieved excellent covariate balance, with all post-IPW standardised mean differences being <0.01. The covariate balance before and after IPW is shown in Supplementary Table S1.

In ordered logistic regression analysis, patient-reported history of carotid stenosis (OR 2.25; 95% CI, 1.38–3.67; *p* < 0.001), right ECA PSV (per 10 cm/s increase: OR 1.31; 95% CI, 1.09–1.57; *p* = 0.004), left ICA PSV (per 10 cm/s increase: OR 1.17; 95% CI, 1.01–1.36; *p* = 0.034), and left ICA stenosis percentage (per 10% increase: OR 1.24; 95% CI, 1.11–1.39; *p* < 0.001) were independently associated with higher CAD burden. The proportional odds assumption was not violated (Brant test *p* = 0.21), and model fit was acceptable (likelihood ratio χ^2^ *p* < 0.001). The main ordered logistic regression model is presented in Table 4. The extended IPW sensitivity model is provided in Supplementary Table S2.

Exploratory ratio-based analyses showed that the ECA/CCA PSV ratio was associated with CAD presence and higher CAD burden, whereas the ICA/CCA ratio showed weaker associations. Specifically, an ECA/CCA ratio ≥ 1.45 was associated with higher CAD burden in adjusted ordered logistic regression (OR 1.52; 95% CI, 1.17–1.98; *p* = 0.002), while an ICA/CCA ratio ≥ 2.0 or ICA occlusion showed a weaker association (OR 1.63; 95% CI, 1.05–2.52; *p* = 0.030). After IPW adjustment, an ECA/CCA ratio ≥ 1.45 remained associated with CAD presence (OR 1.59; *p* = 0.004), with directionally consistent associations across higher CAD burden thresholds. Neither ratio-based index outperformed absolute ECA PSV as a haemodynamic marker of CAD burden. Full exploratory ratio-based results are provided in Supplementary Table S3.

## 4. Discussion

The principal finding of this carotid-focused secondary analysis of the BG Study cohort is that carotid stenosis severity and duplex-derived haemodynamic parameters were associated with increasing angiographic CAD burden. Patient-reported history of carotid stenosis, right external carotid artery (ECA) peak systolic velocity (PSV), left internal carotid artery (ICA) PSV, and left ICA stenosis percentage, identified across the staged statistical analysis, remained independently associated with higher CAD burden in ordered logistic regression. These findings support the concept that carotid duplex ultrasonography reflects not only local extracranial cerebrovascular disease, but also components of systemic atherosclerotic burden.

The association between carotid atherosclerosis and coronary artery disease has been described previously. Prior angiographic studies demonstrated a progressive increase in carotid stenosis prevalence across patients with 0-, 1-, 2-, and 3-vessel CAD, supporting a graded carotid–coronary relationship [3]. This relationship has also been confirmed in systematic review and meta-analytic data showing an association between carotid atherosclerosis and CAD [4]. In patients evaluated for chest pain, carotid disease detected by ultrasonography has been associated with severe CAD [5]. In addition, cohort and meta-analytic data support the prognostic relevance of carotid plaque and intima–media thickness for coronary and cardiovascular outcomes [6,7]. The present study is consistent with the existing literature but extends it by focusing on duplex-derived haemodynamic parameters, particularly ICA and ECA PSV, across angiographic CAD burden categories rather than relying only on plaque, intima–media thickness, or simple present-versus-absent stenosis classifications.

The magnitude of the observed effect sizes is also compatible with previous carotid ultrasound studies. A prior meta-analysis reported a 10–18% increase in cardiovascular risk per 0.1 mm increase in carotid intima–media thickness [6]. Although that work quantified structural arterial wall thickening, whereas the present study assessed haemodynamic and stenotic parameters, both approaches reflect the same pathophysiological continuum of progressive atherosclerosis and increasing vascular resistance. In the present analysis, a 10% increase in carotid stenosis or a 10 cm/s increase in selected carotid velocities was associated with a 17–31% increase in the odds of higher CAD burden. These findings are therefore conceptually consistent with the view that small incremental changes in carotid disease burden correspond to proportional increases in systemic cardiovascular risk.

At the same time, the modest strength of the observed associations is biologically plausible. Coronary atherosclerosis is a multifactorial process influenced by age, sex, diabetes mellitus, renal function, smoking exposure, lipid burden, inflammation, medication use, and referral pathways. Therefore, no single extracoronary vascular marker would be expected to explain CAD burden completely. Earlier angiographic work similarly showed that carotid intima–media thickness correlated significantly but weakly with CAD severity and extent [8].

A distinctive feature of the BG Study cohort is that recruitment occurred during the COVID-19 pandemic and post-pandemic period, when altered referral pathways and heterogeneous cardiovascular presentations increased diagnostic uncertainty [9]. COVID-19-related myocardial injury, myocarditis, Takotsubo syndrome, pulmonary embolism, endothelial dysfunction, and microvascular involvement may mimic or obscure obstructive CAD [10]. This diagnostically heterogeneous cohort therefore provided a real-world test of whether the carotid–coronary association remains detectable under conditions likely to attenuate its strength.

Exploratory ratio-based analyses provided additional haemodynamic context: the ECA/CCA PSV ratio was associated with CAD presence and higher CAD burden, whereas the ICA/CCA ratio showed weaker associations. However, neither ratio-based index outperformed absolute ECA PSV, supporting the primary use of absolute carotid velocities, with ratio-based indices serving as supplementary markers. Prior studies have shown that extracranial cerebrovascular disease in patients with CAD is not limited to the ICA, with ECA and vertebral artery involvement also reported [11]. Therefore, ECA haemodynamics should not be dismissed as a purely secondary finding in systemic vascular assessment.

The practical relevance of these findings may be greatest within vascular surgery pathways. Carotid duplex ultrasonography is widely used to assess extracranial cerebrovascular disease and to guide the management of carotid stenosis, although routine population screening for asymptomatic carotid disease is not recommended [2]. In Germany and similar vascular surgery practice settings, carotid duplex is often performed directly within the specialist assessment pathway, together with peripheral vascular evaluation, and may also be obtained before major non-carotid vascular reconstructions. In such settings, the examination is not used as population screening, but rather as part of a targeted vascular work-up—for example, to exclude clinically relevant bilateral carotid stenosis before physiologically demanding procedures such as aortobifemoral bypass. Therefore, routinely available carotid duplex findings may provide information beyond carotid revascularisation eligibility, including adjunctive signals of systemic atherosclerotic burden.

The cardiovascular vulnerability of carotid and non-carotid vascular surgery patients is illustrated by prospective multicentre data showing peri-operative myocardial injury also after carotid revascularisation [12]. In parallel, routine coronary angiography before carotid endarterectomy has revealed a high prevalence of obstructive coronary lesions [13], and systematic pre-operative coronary angiography followed by selective coronary revascularisation before elective carotid endarterectomy has been associated with improved long-term cardiac outcomes in selected patients [14]. Further studies have demonstrated a high prevalence of silent lesion-specific coronary ischaemia among patients undergoing carotid endarterectomy without known CAD or coronary symptoms, with subsequent work suggesting potential benefit from ischaemia-targeted management in selected patients [15,16]. Together, these studies reinforce the clinical importance of recognising occult coronary vulnerability in selected vascular patients.

Silent CAD and silent coronary ischaemia remain clinically important in vascular patients because symptoms may be absent, underreported, or attributed to reduced mobility, claudication, age, or the known peripheral vascular condition. Silent ischaemia has been highlighted as clinically relevant because it may identify patients at increased cardiovascular risk even in the absence of typical symptoms [17]. In this context, carotid duplex findings should not be regarded as isolated coronary screening tools, but they may contribute to recognising a broader systemic atherosclerotic phenotype in patients already undergoing vascular assessment.

The CARP trial showed no benefit of routine pre-operative coronary revascularisation before elective major vascular surgery in stable patients [18] and contemporary American and European peri-operative guidelines emphasise stepwise cardiovascular assessment rather than universal coronary testing [19,20]. Nevertheless, selected high-risk vascular patients may still benefit from targeted cardiac evaluation when clinical, functional, or vascular imaging findings suggest a high systemic atherosclerotic burden [21]. This is relevant because cardiac complications, including myocardial infarction, remain a major cause of morbidity and mortality after major vascular procedures [22,23].

The observed left–right differences in carotid parameters should be interpreted as exploratory. Left ECA PSV and left ICA stenosis percentage showed stronger unadjusted associations with CAD burden than several right-sided parameters, while right ECA PSV remained independently associated in ordered logistic regression. Potential explanations include side-to-side differences in supra-aortic anatomy, flow patterns, local haemodynamics, and atherosclerotic distribution. The present study was not designed to determine the mechanism of laterality, but these findings may justify further investigation in future cohorts.

## 5. Conclusions

In this carotid-focused secondary analysis of the BG Study cohort, carotid stenosis severity and duplex-derived haemodynamic parameters were modestly but independently associated with increasing angiographic CAD burden. These findings support carotid duplex ultrasonography as a partial marker of systemic atherosclerotic burden, not as a standalone tool for CAD detection or treatment decision-making.

## 6. Limitations

This study has several limitations. First, its single-centre, cross-sectional design limits generalisability and precludes causal inference. Second, the cohort consisted exclusively of patients referred for invasive coronary angiography, introducing selection bias and limiting applicability to unselected vascular surgery or general populations.

Third, CAD burden was assessed anatomically by vessel-count classification based on the number of major coronary vessels with ≥70% stenosis. Quantitative coronary disease scores such as SYNTAX or Gensini could not be calculated because lesion-level angiographic details were not systematically documented in the present dataset. Future studies should evaluate whether carotid duplex-derived haemodynamic markers show stronger associations with quantitative coronary disease scores than with simple vessel-count CAD burden.

Although IPW and ordered logistic regression were used, residual confounding cannot be excluded, particularly from unmeasured factors such as inflammation, medication adherence, frailty, physical activity, socioeconomic status, and treatment intensity. Patient-reported history of carotid stenosis may also have lacked standardised prior Doppler documentation; therefore, the measured duplex parameters obtained during the index hospitalisation remain the primary carotid variables.

Finally, this study did not assess clinical outcomes, peri-operative cardiac events, coronary imaging strategies, or treatment effects. External validation in independent multicentre cohorts, ideally including vascular surgery populations and longitudinal follow-up, is required.

## Figures and Tables

**Table 1:** Baseline characteristics of the study population stratified by coronary artery disease severity.

Variable	No CAD (n = 310)	One-Vessel CAD (n = 190)	Two-Vessel CAD (n = 189)	Three-Vessel CAD (n = 213)	*p*-Value
Age, years	67 (59–76)	68 (57–77)	71 (61–79)	72 (64–79)	<0.001
Body mass index, kg/m^2^	27.4 (24.7–31.0)	27.8 (24.4–31.1)	27.4 (24.7–30.5)	27.3 (24.7–30.4)	0.969
LDL cholesterol, mg/dL	113 (91–150)	100.5 (74.2–140)	105 (75–134)	98 (68–140)	<0.001
HDL cholesterol, mg/dL	54.5 (45–66)	52 (41–63)	48 (40–58)	49 (41–58)	<0.001
Creatinine, mg/dL	0.9 (0.8–1.0)	0.9 (0.8–1.1)	1.0 (0.8–1.2)	1.0 (0.9–1.2)	<0.001
eGFR, mL/min/1.73 m^2^	79 (66–92)	78.5 (67–93)	76 (61–90)	75 (61–89)	0.052
Male sex, n (%)	167 (53.9)	132 (69.5)	146 (77.2)	175 (82.2)	<0.001
Diabetes mellitus, n (%)	68 (21.9)	44 (23.2)	63 (33.3)	84 (39.4)	<0.001
Current smoking, n (%)	172 (55.5)	125 (65.8)	117 (61.9)	145 (68.1)	0.018
Hypertension, n (%)	284 (91.6)	182 (95.8)	187 (98.9)	211 (99.1)	<0.001
Hyperlipidaemia, n (%)	208 (67.1)	94 (49.5)	104 (55.0)	103 (48.4)	<0.001
History of PAD, n (%)	65 (21.0)	50 (26.3)	50 (26.5)	69 (32.4)	0.034
History of stroke, n (%)	26 (8.4)	15 (7.9)	21 (11.1)	15 (7.0)	0.508
Renal insufficiency, n (%)	199 (64.2)	124 (65.3)	128 (67.7)	152 (71.4)	0.358
COPD, n (%)	29 (9.4)	15 (7.9)	16 (8.5)	18 (8.5)	0.95
Statin therapy, n (%)	212 (68.4)	176 (92.6)	172 (91.0)	190 (89.2)	<0.001
Aspirin therapy, n (%)	150 (48.4)	159 (83.7)	153 (81.0)	173 (81.2)	<0.001

Values are presented as median (interquartile range) or n (%). Percentages were calculated within each CAD severity group using available data. Denominators may vary because of missing data. *p*-values were obtained using Kruskal–Wallis test for continuous variables and chi-square test for categorical variables. CAD = coronary artery disease; COPD = chronic obstructive pulmonary disease; eGFR = estimated glomerular filtration rate; PAD = peripheral arterial disease.

**Table 2:** Carotid duplex ultrasound parameters according to coronary artery disease severity.

Variable	No CAD (n = 310)	One-Vessel CAD (n = 190)	Two-Vessel CAD (n = 189)	Three-Vessel CAD (n = 213)	*p*-Value
Measured ICA stenosis of any grade, n (%)	38 (12.3)	42 (22.1)	47 (24.9)	61 (28.6)	<0.001 ^2^
Patient-reported history of carotid stenosis, n (%)	25 (8.1)	28 (14.7)	30 (15.9)	38 (17.8)	0.006 ^2^
Mild ICA stenosis, n (%)	22 (7.1)	17 (8.9)	18 (9.5)	19 (8.9)	0.768 ^2^
Moderate ICA stenosis, n (%)	9 (2.9)	12 (6.3)	14 (7.4)	15 (7.0)	0.089 ^2^
Severe ICA stenosis, n (%)	7 (2.3)	13 (6.8)	15 (7.9)	27 (12.7)	<0.001 ^2^
Left ICA PSV, cm/s	72 (58–96)	78 (61–101)	85 (67–108)	92 (71–118)	0.048 ^1^
Right ICA PSV, cm/s	70 (55–91)	76 (59–97)	82 (64–103)	87 (69–111)	0.018 ^1^
Left ECA PSV, cm/s	85 (68–105)	89 (72–113)	96 (75–118)	104 (80–124)	0.002 ^1^
Right ECA PSV, cm/s	83 (66–103)	90 (71–110)	97 (76–117)	98 (78–121)	0.017 ^1^
Left CCA PSV, cm/s	64 (52–79)	65 (53–82)	66 (54–84)	68 (55–87)	0.276 ^1^
Right CCA PSV, cm/s	63 (51–78)	64 (52–81)	65 (53–83)	66 (54–85)	0.301 ^1^

Values are presented as n (%) or median (interquartile range). Percentages were calculated within each CAD severity group. ^1^ Kruskal–Wallis test for continuous variables. ^2^ Chi-square test across CAD severity groups for categorical variables. Stenosis categories were defined according to NASCET criteria: mild, <50%; moderate, 50–69%; severe, ≥70%. Measured ICA stenosis of any grade refers to any mild, moderate, or severe ICA stenosis identified during index carotid duplex examination. Patient-reported history of carotid stenosis refers to known carotid stenosis before index admission, usually based on prior outpatient ultrasound assessment without standardised spectral Doppler quantification or NASCET-based grading. CAD = coronary artery disease; CCA = common carotid artery; ECA = external carotid artery; ICA = internal carotid artery; PSV = peak systolic velocity.

**Table 3:** Post hoc pairwise intergroup comparisons of carotid duplex parameters across CAD severity groups.

Variable	Global *p*-Value	Significant Pairwise Comparison	Adjusted *p*-Value
Measured ICA stenosis of any grade	<0.001 ***	No CAD vs. 1VD	0.033 *
Measured ICA stenosis of any grade	<0.001 ***	No CAD vs. 2VD	0.002 **
Measured ICA stenosis of any grade	<0.001 ***	No CAD vs. 3VD	<0.001 ***
Patient-reported history of carotid stenosis	0.006 **	No CAD vs. 2VD	0.048 *
Patient-reported history of carotid stenosis	0.006 **	No CAD vs. 3VD	0.006 **
Severe ICA stenosis	<0.001 ***	No CAD vs. 2VD	0.033 *
Severe ICA stenosis	<0.001 ***	No CAD vs. 3VD	<0.001 ***
Left ECA PSV	0.002 **	No CAD vs. 3VD	0.002 **
Right ECA PSV	0.017 *	No CAD vs. 3VD	0.042 *

Only statistically significant post hoc pairwise comparisons are displayed. Global intergroup comparisons across the four CAD severity groups were assessed using the chi-square test for categorical variables and the Kruskal–Wallis test for continuous PSV variables. For categorical variables, significant global tests were followed by pairwise Fisher’s exact tests with Bonferroni correction for multiple comparisons. For continuous PSV variables, significant global tests were followed by Dunn’s post hoc test with Holm correction. Adjusted *p*-values are reported. Significance levels are indicated as * *p* < 0.05, ** *p* < 0.01, and *** *p* < 0.001. CAD = coronary artery disease; ECA = external carotid artery; ICA = internal carotid artery; PSV = peak systolic velocity; 1VD = one-vessel CAD; 2VD = two-vessel CAD; 3VD = three-vessel CAD.

**Table 4:** Multivariable ordered logistic regression model for association between carotid parameters and CAD burden.

Predictor	OR (95% CI)	*p*-Value
Patient-reported history of carotid stenosis †	2.25 (1.38–3.67)	<0.001
Right ECA PSV, per 10 cm/s ‡	1.31 (1.09–1.57)	0.004
Left ICA PSV, per 10 cm/s ‡	1.17 (1.01–1.36)	0.034
Left ICA stenosis, per 10% §	1.24 (1.11–1.39)	<0.001

Model type: multivariable ordered logistic regression with CAD burden as ordinal outcome: no CAD, one-vessel disease, two-vessel disease, and three-vessel disease. The proportional odds assumption was met using the global Brant test (*p* = 0.21). McFadden pseudo R^2^ = 0.074; approximate Nagelkerke R^2^ = 0.08; likelihood ratio χ^2^(18) = 75.3, *p* < 0.001; N = 902. † Reference category = no patient-reported history of carotid stenosis. ‡ PSV = peak systolic velocity in cm/s, scaled per 10 cm/s increase. § ICA stenosis measured as percentage diameter reduction, scaled per 10% increase. CAD = coronary artery disease; CI = confidence interval; ECA = external carotid artery; ICA = internal carotid artery; OR = odds ratio; PSV = peak systolic velocity.

## Data Availability

The datasets generated and/or analysed during the current study are not publicly available due to data protection and privacy regulations governing patient information in Germany, but are available from the corresponding author upon reasonable request and in accordance with institutional and ethical guidelines.

## Supplementary Materials

The following supporting information can be downloaded at https://www.mdpi.com/article/10.3390/jcm15114383/s1. Table S1. Covariate balance before and after inverse probability weighting. Table S2. Sensitivity analysis using extended IPW-weighted ordered logistic regression model. Table S3. Exploratory ICA/CCA and ECA/CCA PSV ratio-based analyses.
