# Supplementary material for: Carotid Duplex-Derived Markers Across Angiographic Coronary Artery Disease Burden: A Pandemic-Era Real-World Cohort Study
Source: J Clin Med. 2026 Jun 5;15(11):4383. doi: 10.3390/jcm15114383 (PMC13257789; doi:10.3390/jcm15114383)
Supplement: Supplementary file 1 [file jcm-15-04383-s001.zip › jcm-4290561-supplementary.pdf]

Supplementary Table S1. Covariate balance before and after inverse probability weighting.

Covariate	SMD Before IPW	SMD After IPW
Age, years	0.212	<0.01
Male sex	0.134	<0.01
Diabetes mellitus	0.157	<0.01
Hypertension	0.098	<0.01
LDL cholesterol	0.142	<0.01
Creatinine	0.121	<0.01
eGFR	0.115	<0.01
Smoking status	0.165	<0.01

SMD = standardised mean difference; IPW = inverse probability weighting; eGFR = estimated glomerular filtration rate. Good covariate balance was defined as SMD <0.10. Propensity scores were estimated using age, sex, diabetes mellitus, hypertension, smoking status, LDL cholesterol, creatinine, and eGFR.

Supplementary Table S2. Sensitivity analysis using extended IPW-weighted ordered logistic regression model.

Variable	OR	95% CI	*p*-Value
Patient-reported history of carotid stenosis †	2.18	1.33–3.58	<0.001
Right ECA PSV, per 10 cm/s	1.29	1.07–1.55	0.006
Left ICA PSV, per 10 cm/s	1.16	1.00–1.34	0.048
Left ICA stenosis, per 10%	1.23	1.10–1.38	<0.001

Extended inverse probability-weighted ordered logistic regression model with coronary artery disease burden as the ordinal outcome: no coronary artery disease, one-vessel disease, two-vessel disease, and three-vessel disease. The extended model included age, sex, diabetes mellitus, hypertension, smoking status, LDL cholesterol, creatinine, and estimated glomerular filtration rate. All *p*-values are two-sided. † Reference category = no patient-reported history of carotid stenosis. CI = confidence interval; ECA = external carotid artery; ICA = internal carotid artery; OR = odds ratio; PSV = peak systolic velocity.

Supplementary Table S3. Exploratory ICA/CCA and ECA/CCA PSV ratio-based analyses.

Marker	Analysis	Result	Interpretation
Max ICA/CCA ratio, continuous	CAD burden correlation	$\rho = 0.080$; $p = 0.017$	Weak association; limited CAD discrimination
Max ICA/CCA ratio ≥2.0 or ICA occlusion	Adjusted OLR for CAD burden	OR 1.63 (1.05–2.52); $p = 0.030$	Supplementary stenosis-related signal
Max ECA/CCA ratio, continuous	CAD burden correlation	$\rho = 0.157$; $p < 0.001$	Weak-to-modest association
Max ECA/CCA ratio ≥1.45	Adjusted logistic regression for CAD presence	OR 1.66 (1.19–2.32); $p = 0.003$	Associated with CAD presence
Max ECA/CCA ratio ≥1.45	Adjusted OLR for CAD burden	OR 1.52 (1.17–1.98); $p = 0.002$	Associated with higher CAD burden
Max ECA/CCA ratio ≥1.45	IPW-weighted CAD presence model	OR 1.59; $p = 0.004$	Association persisted after IPW

Ratio-based indices were exploratory sensitivity analyses. The ICA/CCA ratio is not applicable in complete ICA occlusion; therefore, ICA occlusion was analysed as a separate high-risk category. Absolute ECA PSV from the main model was used as the reference haemodynamic marker; neither ratio-based index outperformed absolute ECA PSV. CAD = coronary artery disease; CCA = common carotid artery; ECA = external carotid artery; ICA = internal carotid artery; IPW = inverse probability weighting; OLR = ordered logistic regression; OR = odds ratio; PSV = peak systolic velocity.
